# Ethnic differences in etiologies and audiological outcomes of cochlear implantation in Israeli children

**DOI:** 10.1007/s00405-025-09938-0

**Published:** 2025-12-22

**Authors:** Yael Levi, Shanit Rich, Rema Jbarah, Salma Khoury Shoufani, Michal Kaufmann Yehezkely, Sagit Stern Shavit

**Affiliations:** 1 Faculty of Medicine, Hebrew University, Jerusalem, Israel; 2 Speech & Hearing Center, Hadassah Medical Center, Jerusalem, Israel; 3 Department of Communication Disorders, Jerusalem Multidisciplinary College, Jerusalem, Israel; 4 Department of Otolaryngology/Head & Neck Surgery, Hadassah Medical Center, Jerusalem, Israel

**Keywords:** Cochlear implants, Pediatric hearing loss, Ethnic disparities, Hearing loss etiology, Hearing outcomes

## Abstract

**Purpose:**

To examine differences in hearing loss etiologies and cochlear implant outcomes between Jewish and Arab pediatric populations in Israel, addressing the understudied influence of ethnic disparities.

**Methods:**

A retrospective cohort study was conducted on 120 children (53 Jewish, 67 Arab) who underwent 171 cochlear implantations at our hospital between May 2018 and December 2022. Data included demographics, socioeconomic status, etiology, and audiological outcomes. Outcomes were assessed using pure tone averages (PTA) and speech thresholds (SRT/SDT) at seven-time points over 60 months. Predictors of early and long-term outcomes were evaluated using multivariate ANCOVA models.

**Results:**

Jewish and Arab children had comparable demographics, but significant socioeconomic differences (399.5 ± 378.7 vs. 110.3 ± 181.6, *p* < 0.0001). Hearing loss etiologies differed significantly: Arab children showed predominantly congenital causes (68.7%), mainly genetic (62.7%), while Jewish children had higher acquired causes (54.7%), primarily CMV-related (52.5%). PTA thresholds were significantly better in Jewish patients at 1–4, 25–36, and 49–60 months post-implantation (*p* < 0.05). SRT/SDT thresholds did not differ. Multivariate analysis identified ethnicity, younger age at surgery, primary implantation status, and etiology as significant predictors of outcomes. Socioeconomic rank was not independently associated with outcomes.

**Conclusion:**

Ethnic differences in cochlear implant outcomes persist despite Israel’s universal healthcare system, reflecting the impact of unmeasured social, cultural, and behavioral factors. These findings underscore the importance of individualized treatment strategies that consider both biological and sociocultural factors to optimize outcomes for all populations.

## Introduction

Cochlear implants have become a standard treatment for hearing rehabilitation in children and adults with severe to profound sensorineural hearing loss. However, outcomes vary across individuals and depend on numerous known and unknown factors, particularly in pediatric populations, where results reflect the complex interplay of individual and familial factors [1]. Research has extensively studied the factors that influence cochlear implant outcomes, assessing success through various measures, including language acquisition, verbal communication skills, and performance on sentence or speech perception tests. Key factors shown to affect outcomes include early age at implantation, particularly before 12 months, which is crucial for optimal speech and language development comparable to normal-hearing peers [2–4]. Bilateral implantation has also demonstrated superior results compared to unilateral implantation in children with bilateral deafness [1]. The presence and number of comorbidities, specifically language and communication disorders, followed by cognitive impairments and autism spectrum disorders, have a strong correlation with post-implantation outcomes [5]. Anatomical variations in the hearing system can also impact results, depending on the type of anomaly and associated impairments [1].

Socioeconomic factors have also been shown to influence cochlear implant outcomes, primarily through disparities in access to and utilization of post-implantation rehabilitation services [6]. Lower socioeconomic status has been associated with poorer language comprehension [7], while parental educational level has been shown to affect language abilities and sentence comprehension [8]. A systematic review, encompassing 45 studies published between 1999 and 2021, revealed that socioeconomic disparities affect cochlear implant outcomes; however, there remains insufficient evidence regarding the impact of race or ethnicity [6]. Indeed, the influence of racial and ethnic disparities on cochlear implant outcomes remains understudied globally, and particularly within Israel.

The population composition in Israel is both unique and diverse. As of 2025, Jewish citizens comprised approximately 78.5% of the population, while Arab citizens made up about 21.5% [9]. In Jerusalem, this distribution was approximately 57% Jewish and 38% Arab [10].

The Arab population in Israel faces significant socioeconomic disparities. On average, Arab households earn 37% less than Jewish households and have a 47% lower standardized per capita income. Arab neighborhoods predominantly rank in the lowest socioeconomic clusters (1–3 on a 10-point scale), whereas Jewish neighborhoods typically rank between 4 and 9. These disparities are similarly evident in Jerusalem [11].

Our hospital serves a unique, diverse patient population compared to most hospitals in Israel, making it particularly suitable for comparative research between these two ethnic groups. Several Israeli studies have reported medical differences between Jewish and Arab populations. For example, Arab patients with Colorectal cancer tend to be younger and are more often diagnosed with advanced-stage [12]. Arabs have a higher proportion of extracapsular fractures among hip fractures, though outcomes were similar across groups [13]. Studies of autism spectrum disorder revealed ethnic-specific barriers to diagnosis, particularly among the Bedouin population [14].

Given the differences between these populations, it is plausible that cochlear implantation outcomes may also vary. Therefore, this study aims to examine the differences in the etiologies and outcomes of cochlear implants between Jewish and Arab children treated at our hospital, with the goal of identifying disparities and developing targeted interventions to improve outcomes across all populations.

## Methods

In this retrospective cohort study, data were collected from the electronic medical records of the hospital for all children (0–14 years) who underwent cochlear implantation between May 2018 and December 2022, with a minimum follow-up of one year. The upper age limit was set at 14 years, as older adolescents follow adult rehabilitation protocols and the small number of implantations in this group precluded meaningful analysis. Patients were excluded if they had severe cognitive conditions that could impact assessment, if data were missing, or if they experienced a hard or soft implant failure. Patients who underwent reimplantation following implant failure were included in the research and categorized as reimplantation cases.

Collected data included patient demographics, including ethnicity, age at implantation, and sex. Ethnicity (Jewish or Arab) was determined based on documentation in the electronic medical record. The terms ‘Jewish’ and ‘Arab’ represent broad ethnic-cultural categories consistent with Israeli demographic classifications. The Arab population in our study was predominantly from East Jerusalem and therefore consisted mainly of Muslim patients. The Jewish population in Israel is characterized by extensive admixture across generations; thus, we did not differentiate between Jewish subgroups in our analysis. Socioeconomic status was extrapolated from the socioeconomic classification of municipalities and local councils in Israel, as well as the socioeconomic ranking of Jerusalem neighborhoods, based on data from the Israel Central Bureau of Statistics [15]. The socioeconomic index (scale 1–1629, where lower scores indicate lower socioeconomic status) incorporates 14 variables across demographics, education, employment, and standard of living, including household income, housing density, educational attainment, and employment rates. Each patient was assigned a socioeconomic rank based on their home address. For localities with multiple statistical areas, the mean ranking across all areas within that city or neighborhood was used.

Implant characteristics included the implant manufacturer and whether the implantation was primary, consecutive, or a reimplantation. Clinical information encompassed hearing loss etiology, baseline hearing status, presence of comorbidities, surgical complications, and intraoperative test results.

Hearing loss etiologies were classified as congenital or acquired. Congenital causes included non-syndromic genetic mutations, syndromic hearing loss, inner ear anomalies, and auditory neuropathy. Acquired causes included congenital cytomegalovirus (cCMV) infection, trauma, prenatal complications, and meningitis.

Outcomes were measured using pure-tone averages (PTA) and speech reception thresholds (SRT), or speech detection thresholds (SDT) in cases where SRT could not be completed. When both tests were available, SRT was used for analysis. Audiological measurements were collected at seven post-implantation intervals (1–4 months, 5–8 months, 9–12 months, 13–24 months, 25–36 months, 37–48 months, and 49–60 months) to accommodate variable follow-up patterns, as not all children were tested at each interval due to irregular attendance and COVID-19–related disruptions. A repeated-measures or mixed-effects model was not feasible due to unbalanced and incomplete longitudinal data. For each time interval, if multiple test results were available, the best performance was selected for analysis.

Demographic characteristics, clinical data, and audiological outcomes were compared between Arab and Jewish patients. For demographic analyses, each child was counted once, whereas for implant-level analyses, each implantation was counted separately, treating bilateral and reimplantation cases as independent observations. The study was approved by the hospital Ethics Committee (0160-23-HMO) with a waiver of informed consent.

### Statistical methods

Statistical analysis was performed using the Chi-square test or the Fisher-Freeman-Halton Exact Test for categorical variables. For continuous variables, either the two-sample t-test or the Mann-Whitney nonparametric test was used. Comparing continuous variables between three independent groups was performed by using the Kruskal-Wallis nonparametric test. Assessing the strength of the association between two continuous variables was performed by calculating the Pearson correlation coefficient or the Spearman nonparametric correlation coefficient. Nonparametric tests were used in cases with many missing data values and a non-normal distribution. The Multivariate ANCOVA model was applied to simultaneously assess the effect of multiple independent variables on a continuous dependent variable.

All statistical tests were two-tailed, and a p-value ≤ 0.05 was considered statistically significant. All statistical analyses were performed using SPSS version 30 (IBM Corp., Armonk, NY, USA).

## Results

**Fig. 1:**
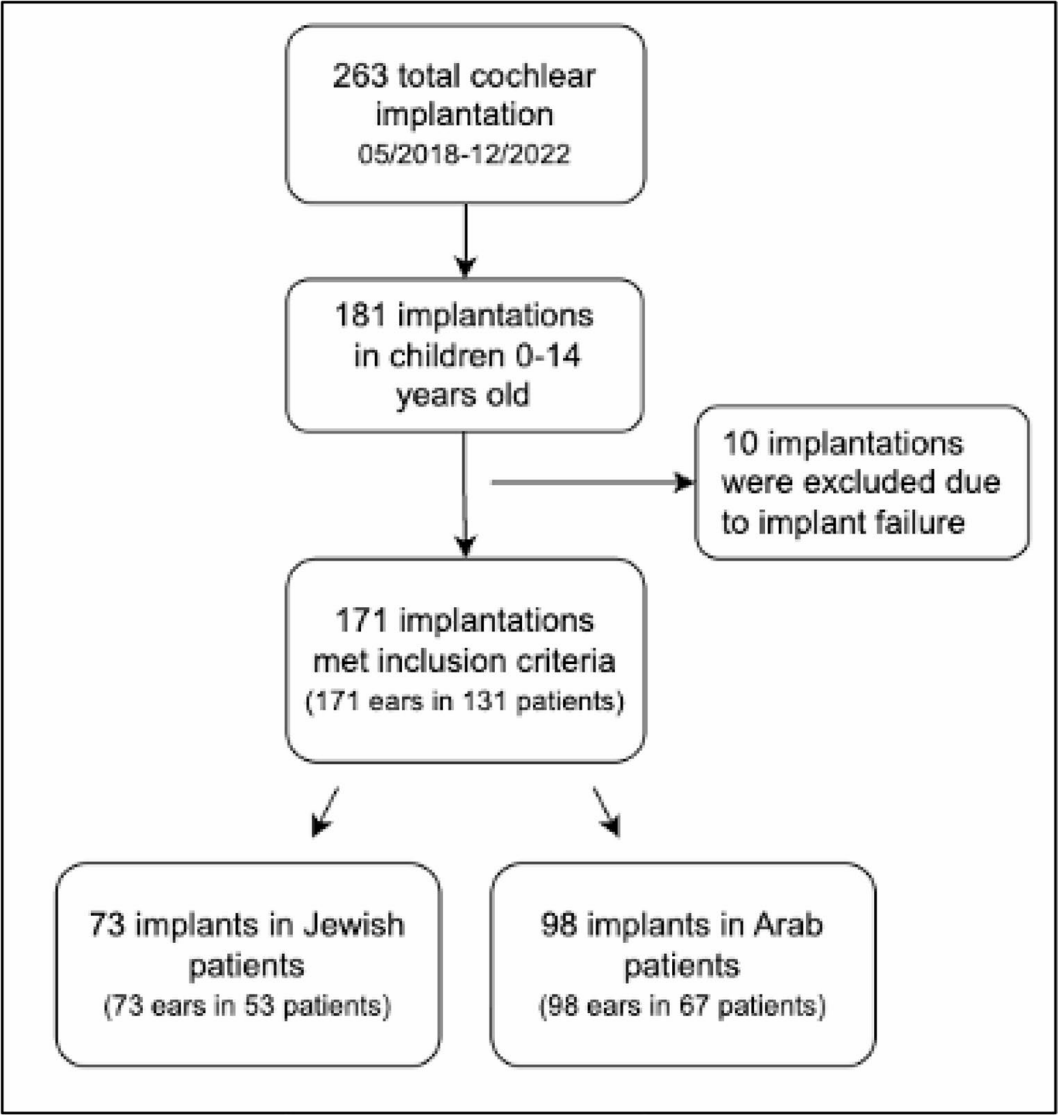
Patient selection flowchart. Of 263 cochlear implantations performed between May 2018 and December 2022, 181 were in children aged 0–14. After excluding 10 implant failures, 171 implantations (in 120 children: 53 Jewish, 67 Arab) met the inclusion criteria.

Between May 2018 and December 2022, 263 cochlear implantations were performed at our tertiary medical Center. Of these, 181 procedures were done in children aged 0–14 years. Ten implantations were excluded due to implant failure. The final cohort included 171 implantations in 120 children, of whom 53 were Jewish, and 67 were Arab (Fig. 1).

### Demographic, audiological, and surgical characteristics

Analysis of 120 children (53 Jewish and 67 Arab) demonstrated comparable demographic and clinical characteristics between the two ethnic groups, including age at surgery, sex distribution, and comorbidity rates (Table 1). Socioeconomic status differed significantly between groups (399.5 ± 378.7 vs. 110.3 ± 181.6, *p* < 0.0001), indicating a higher socioeconomic classification among Jewish participants.

**Table 1:** Demographic and clinical characteristics by ethnicity

Characteristic	Total (*N* = 120)	Jewish (*n* = 53)	Arab (*n* = 67)	*p*-value
**Age (years)¹**	4.17 ± 3.72	4.0 ± 3.7	3.5 ± 3.4	0.371
**Sex²**				0.494
Male	66 (55.0)	31 (58.5)	35 (52.2)	
Female	54 (45.0)	22 (41.5)	32 (47.8)	
**Any Comorbidity²**				0.233
No	88 (73.3)	36 (67.9)	52 (77.6)	
Yes	32 (26.7)	17 (32.1)	15 (22.4)	
**Socioeconomic rank (1–1**,**629)¹**	248.3 ± 325.2	399.5 ± 378.7	110.3 ± 181.6	< 0.001*

Values are presented as:

¹Mean ± Standard Deviation

²n (%)

*Statistically significant (*p* < 0.05)

Analysis of 171 implanted ears (73 Jewish, 98 Arab) revealed comparable surgical and audiological parameters (Table 2). In both populations, the majority of implantations were primary procedures. Profound hearing loss was the most common diagnosis, and in over 97% of cases, electrode insertion was completed without complications. Cochlear-brand implants were most frequently used in both groups. However, the distribution of manufacturers differed significantly between populations: Med-El devices were more common among Arab patients (43.9% vs. 15.1%), while Advanced Bionics devices were more prevalent among Jewish patients (23.3% vs. 5.1%; *p* < 0.001).

**Table 2:** Surgical and Device-Related characteristics

Characteristic	Total (*N* = 171)	Jewish (*n* = 73)	Arab (*n* = 98)	*p*-value
**First CI²**				0.136
Yes (primary implantation)	142 (83.0)	57 (78.1)	85 (86.7)	
No (second ear implants and reimplantation)	29 (17.0)	16 (21.9)	13 (13.3)	
**Hearing Impairment²**				0.205
Moderate	5 (3.0)	4 (5.6)	1 (1.1)	
Severe	5 (3.0)	3 (4.2)	2 (2.1)	
Severe to Profound	26 (15.6)	13 (18.1)	13 (13.7)	
Profound	131 (78.4)	52 (72.2)	79 (83.2)	
**Insertion²**				0.576
Normal	168 (98.2)	71 (97.3)	97 (99.0)	
Difficult	3 (1.8)	2 (2.7)	1 (1.0)	
**Company of Implant²**				< 0.001*
Advanced Bionics	22 (12.9)	17 (23.3)	5 (5.1)	
Cochlear	95 (55.6)	45 (61.6)	50 (51.0)	
Med-El	54 (31.6)	11 (15.1)	43 (43.9)	

Values are presented as:

¹Mean ± Standard Deviation

²n (%)

*Statistically significant (*p* < 0.05)

### Etiology of hearing loss

The etiology of hearing loss was significantly different between the two ethnic groups. The proportion of patients with unknown etiology was similar in both groups (Jewish: 24.5%, Arab: 23.9%). However, among those with a known etiology, the distribution of causes varied (Table 3). Arab children demonstrated predominantly congenital hearing loss (68.7%), with genetic causes as the primary etiology (62.7% of known causes). In contrast, Jewish children exhibited higher rates of acquired hearing loss (54.7%), with CMV as the predominant cause (52.5% of known causes). Other etiologies also showed ethnic-specific trends: inner ear anomalies were more frequently diagnosed among Arab children (15.7% vs. 7.5%), while meningitis-related hearing loss was observed exclusively among Jewish patients (10.0%).

**Table 3:** Etiology of hearing loss

	Total (*N* = 120)	Jewish (*n* = 53)	Arab (*n* = 67)	*p*-value
**Etiology**²				< 0.001*
Unknown	29 (24.2)	13 (24.5)	16 (23.9)	
Congenital	57 (47.5)	11 (20.8)	46 (68.7)	
Acquired	34 (28.3)	29 (54.7)	5 (7.5)	
**Detailed Etiology²**				< 0.001*
Total known	91 (100.0)	40 (100.0)	51 (100.0)	
CMV	22 (24.2)	21 (52.5)	1 (2.0)	
Inner ear anomaly	11 (12.1)	3 (7.5)	8 (15.7)	
Genetic (non-syndromic)	36 (39.6)	4 (10.0)	32 (62.7)	
Genetic (syndromic)	7 (7.7)	2 (5.0)	5 (9.8)	
Meningitis	4 (4.4)	4 (10.0)	0 (0.0)	
Auditory Neuropathy	3 (3.3)	2 (5.0)	1 (2.0)	
Prenatal Complications	6 (6.6)	2 (5.0)	4 (7.8)	
Trauma	2 (2.2)	2 (5.0)	0 (0.0)	

Values are presented as n (%)

*Statistically significant (*p* < 0.05)

### Audiological outcomes

**Fig. 2:**
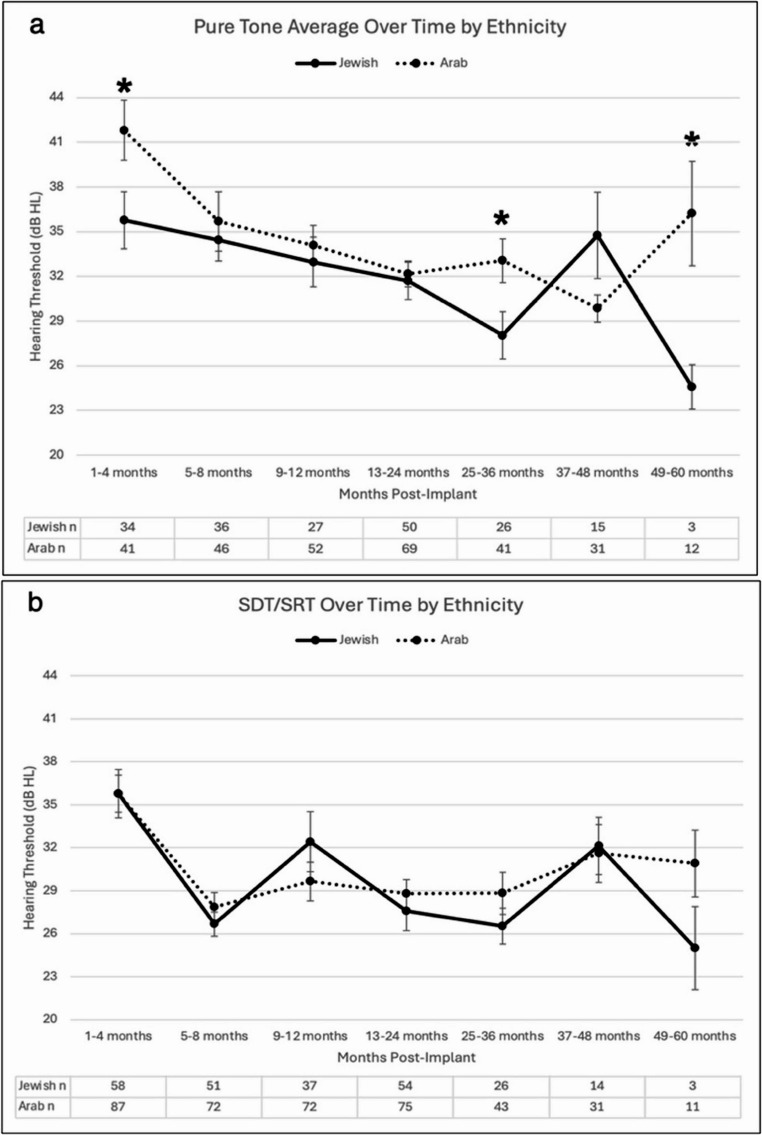
Audiological outcomes over time by ethnicity. (**2a**) PTA values (dB) over 60 months post-implantation show significantly better thresholds in Jewish patients at 1–4, 25–36, and 49–60 months (*p < 0.05). (**2b**) SDT/SRT values showed no significant differences between groups at any time point. Error bars represent the standard error of the mean. Lower values indicate better hearing thresholds.

Audiological outcomes analysis over a 60-month follow-up revealed significant differences in pure tone averages between the two ethnic groups at three time points (Fig. 2a). Jewish patients demonstrated significantly better PTA thresholds at 1–4 months post-implantation (35.77 ± 11.19 vs. 41.81 ± 12.89 dB, *p* = 0.018), 25–36 months (28.04 ± 8.06 vs. 33.05 ± 9.34 dB, *p* = 0.014), and at 49–60 months (24.58 ± 2.60 vs. 36.22 ± 12.10 dB, *p* = 0.041). Due to smaller sample sizes at the last time point, non-parametric tests were used. In contrast, no statistically significant differences were observed between the two groups in SRT/SDT thresholds at any time point (Fig. 2b). Both groups demonstrated gradual improvement in hearing thresholds over time, with the most notable improvements occurring within the first 24 months following implantation.

### Predictors of audiological outcomes

**Fig. 3:**
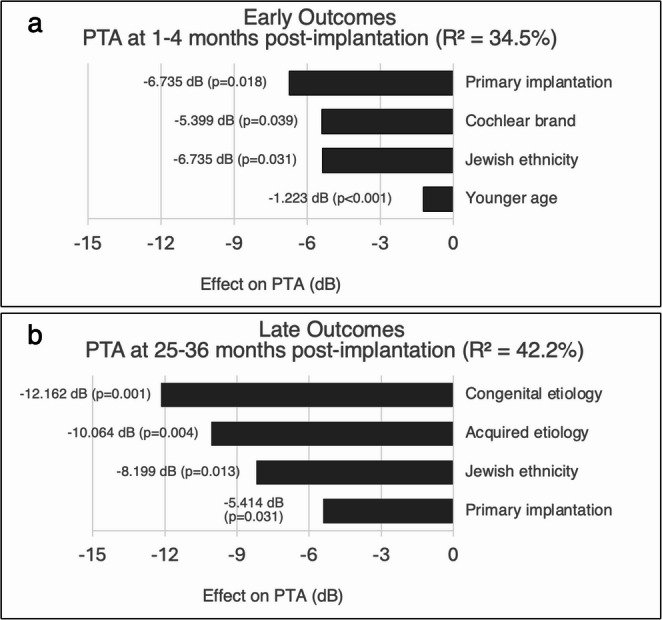
Predictors of audiological outcomes at early and late time points. (**3a**) At 1–4 months post-implantation, younger age, primary implantation, Jewish ethnicity, and Cochlear-brand devices were associated with better PTA outcomes (R² = 34.5%). (**3b**) At 25–36 months, congenital and acquired etiologies, Jewish ethnicity, and primary implantation predicted better outcomes (R² = 42.2%). Negative values indicate better hearing thresholds (lower PTA).

To explore variables associated with early post-implantation outcomes, we analyzed factors influencing PTA at 1–4 months. In univariate analysis, younger age at surgery, Jewish ethnicity, Cochlear manufacture, primary implantation, and profound hearing loss were significantly associated with better hearing thresholds (Table 4). These variables were entered into a multivariate ANCOVA model, which explained 34.5% of the variance in outcomes. In this model, younger age at surgery and primary implantation remained significant predictors of better PTA. Ethnicity also remained independently associated with improved performance, while Cochlear-brand devices showed a modest advantage (Fig. 3a).

**Table 4:** Multivariate analysis of factors affecting pure tone averages

	Early Outcomes (1–4 months) Jewish *n* = 17, Arab *n* = 16			Long-term Outcomes (25–36 months) Jewish *n* = 17, Arab *n* = 16		
	Univariate	Multivariate		Univariate	Multivariate	
Variable	Correlation to Early Outcomes	β Coeff	p-value	Correlation to Late Outcomes	β Coeff	p-value
Age at surgery (years)	Significant	−1.223	**< 0.001**	Significant	-	> 0.05
Ethnicity (Jewish vs. Arab)	Significant	5.383	**0.031**	Significant	8.199	**0.013**
Primary CI (vs. consecutive/reimplantation)	Significant	−6.735	**0.018**	Significant	−5.414	**0.031**
Company-Cochlear (vs. others)	Significant	−5.399	**0.039**	Significant	-	> 0.05
Profound hearing loss	Significant	-	> 0.05	NS		
Etiology - Congenital (vs. unknown)	NS			Significant	−12.162	**0.001**
Etiology - Acquired (vs. unknown)	NS			Significant	−10.064	**0.004**
Socioeconomic rank	NS			Significant	-	> 0.05
Sex	NS			NS		
Comorbidities	NS			NS		

1. Univariate analysis: Pearson or Spearman correlation as appropriate

2. Multivariate analysis performed using stepwise ANCOVA

3. NS = Not significant

4. Negative β coefficients indicate better hearing outcomes (lower PTA values)

5. P-values < 0.05 considered statistically significant

6. PTA = Pure Tone Average; CI = Cochlear Implant

For long-term outcomes at 25–36 months, a different pattern emerged. Univariate analysis identified etiology, socioeconomic rank, implant type, ethnicity, and age at surgery as potential predictors (Table 4). In the multivariate model, which explained 42.2% of the outcome variance, etiology was the strongest predictor: both congenital and acquired causes were associated with better outcomes than those with an unknown etiology. Primary implantation and ethnicity remained significant, with Jewish patients continuing to demonstrate better hearing thresholds (Fig. 3b). Other factors, including age at surgery and socioeconomic rank, did not retain significance after adjustment.

## Discussion

Medical differences between Jewish and Arab populations in Israel have been previously demonstrated [12–14]. Our study aimed to identify similar disparities in CI outcomes between Jewish and Arab pediatric patients treated at our medical center. We found significant ethnic differences in the etiology of hearing loss and audiological outcomes at multiple time points.

Whereas both groups were comparable in sex, comorbidities, and age at implantation, Jewish patients demonstrated superior PTA at 1–4 months, 25–36 months, and 49–60 months post-implantation. However, speech reception/detection thresholds remained similar throughout follow-up. Cochlear implant outcomes can be evaluated through audiological measures (pure tone audiometry, speech audiometry) or patient-reported outcome measures (PROMs). While audiological measures provide quantifiable data, they may not fully capture real-world communication abilities. In contrast, PROMs can be influenced by patient perception and are less reliable in young children [16]. We selected PTA and SRT/SDT given our young pediatric cohort and limited questionnaire data availability.

The observed PTA differences between groups ranged from 5 to 6 dB at early and intermediate time points to approximately 12 dB at 49 to 60 months. Although statistically significant, Le Prell et al. noted that differences of 5 dB in pure tone audiometry generally fall within test-retest variability in young children, where behavioral thresholds are challenging to obtain [17]. The similarity in speech thresholds, despite these modest PTA differences, suggests that functional communication abilities may be more comparable between groups than pure tone measures alone imply. Thus, the observed audiometric differences may not translate into meaningful variations in everyday hearing performance. Future studies incorporating speech-language development and quality-of-life measures are needed to clarify the functional significance of these findings.

Multivariate analysis was conducted to identify variables predicting better audiological outcomes at various time points. Ethnicity remained a consistent predictor, with Jewish patients showing better PTA even after adjusting for confounders. Although socioeconomic status differed significantly between Jewish and Arab children (mean rank 409.4 vs. 107.3, *p* < 0.0001), it was not a significant predictor at any time.

This finding contrasts with a recent meta-analysis by Mueller et al., which found that children from racial and ethnic minority groups had worse speech recognition outcomes and receptive language scores compared to white children, with socioeconomic status and parental education playing major roles [18]. Similarly, a UK study spanning 20 years found that children for whom English was an Additional Language (EAL) demonstrated poorer long-term language outcomes than their native English-speaking peers, with home language, socioeconomic deprivation, and parental education predicting post-implantation abilities [19]. A Norwegian cohort similarly showed lower speech recognition scores among children born to non-Nordic parents despite equal access to cochlear implants [20].In our study, both Jewish and Arab children are native populations within Israel; however, Hebrew functions as a second language for most Arab children.

The lack of an independent effect of socioeconomic status on outcomes in our study, despite the significant disparity between groups, contrasts with findings from other healthcare systems and warrants careful interpretation. Israel’s universal healthcare system ensures equitable access to cochlear implantation and standardized post-operative care, which may mitigate socioeconomic disparities observed elsewhere. Nevertheless, the persistence of ethnicity as a predictor suggests the influence of unmeasured social, cultural, or behavioral factors, such as consistency of device use, participation in rehabilitation, adherence to follow-up, and family engagement in auditory training. Area-based socioeconomic measures may also fail to adequately capture individual-level differences in household income, parental education, or language environment. Additional contributors, including genetic heterogeneity and pre-operative auditory experience, merit future investigation to clarify the mechanisms underlying these ethnic differences.

The most notable distinction between the two groups was in the etiology of hearing loss. Arab children predominantly had congenital hearing loss (68.7%), mostly genetic (62.7% of known causes). This difference may be partly explained by the higher rates of consanguineous marriage in Israeli Arab populations (42% in Muslims, 22% in Christians, 47% in Druze) [21], which increase the prevalence of autosomal recessive disorders, including hearing loss [22]. In contrast, Jewish children had higher rates of acquired hearing loss (54.7%), mainly CMV-related (52.5%), possibly related to lower CMV seroprevalence among Jewish women at pregnancy onset (about 60% vs. >90% in Arab women), resulting in greater susceptibility to primary CMV infection and its associated risk of congenital hearing loss [23]. These distinct etiological patterns have important prognostic implications. Etiology was identified as the strongest predictor of outcomes at 25–36 months, with congenital causes associated with better outcomes. This aligns with literature demonstrating that genetic causes of deafness, particularly when cochlear implantation is performed early, are associated with superior cochlear implant outcomes compared to acquired etiologies [24–26]. Nevertheless, it does not explain the favorable PTA seen in Jewish children, who mostly had acquired hearing loss, suggesting that the advantages of genetic hearing loss can be outweighed by other ethnicity-related factors, healthcare utilization, rehabilitation participation, and unmeasured social and cultural factors.

Additional factors, unrelated to ethnicity, also predicted CI outcomes in our model. Younger age at surgery was a strong predictor of better PTA (1–4 months), consistent with the known critical period for auditory development and neural plasticity [27–29]. Primary implantation status was associated with better outcomes at both early and long-term intervals, likely due to the absence of delays or interruptions in auditory input, which can negatively impact outcomes [30]. The device manufacturer influenced early outcomes, with an advantage for devices from the Cochlear brand implants. However, this finding should be interpreted cautiously, given the lack of clear evidence for superiority in the literature. Additionally, differences in health insurance companies with different preferred cochlear implant suppliers may influence manufacturer selection, potentially confounding these results and contributing to the observed distribution patterns. Device brand was not a significant factor for long-term outcomes.

Our study has several limitations. As a single-center, retrospective cohort study, it is susceptible to selection bias, missing data, and may not accurately represent other medical centers. Nevertheless, the unique population distribution in Jerusalem, combined with our substantial experience with both populations, provides meaningful insights to highlight disparities in CI outcomes in children. Implant-level analyses treated each implantation as independent, without fully accounting for correlations between bilateral or reimplanted ears; however, this likely affected descriptive rather than inferential analyses of ethnic differences. Reliance on audiometric measures (PTA and SRT/SDT) without systematic assessment of speech-language, educational, or quality-of-life outcomes limits interpretation of whether audiometric differences reflect real-world functional disparities. Other limitations include a relatively short follow-up period that may not capture the entire benefit trajectory, as well as the absence of data on intra-group ethnic variation, family structure, rehabilitation adherence, device use, and cultural attitudes. Individual-level socioeconomic information (parental education, occupation, household income) was also unavailable, restricting evaluation beyond neighborhood-level classification. Finally, time-point–stratified analyses were conducted without correction for multiple comparisons, reflecting hypothesis-driven evaluation of correlated developmental outcomes. Based on our preliminary findings, our future studies will aim to include comprehensive cultural data to better understand and address these disparities.

## Conclusion

This study demonstrates that cochlear implantation provides significant hearing benefit to both Jewish and Arab pediatric populations, despite notable differences in socioeconomic status. Notable differences were found in the etiology of hearing loss, with PTA favoring Jewish patients at several time points. Other predictors of outcome included younger age at surgery, primary implantation status, and device manufacturer. The persistence of ethnic differences in outcomes, even after controlling for known confounders, likely reflects modifiable social and environmental factors rather than inherent ethnic differences. These findings underscore the importance of individualized, culturally responsive care that incorporates etiological and socioeconomic factors, prioritizes linguistically accessible family education, promotes consistent device use, and ensures equitable access to community-based rehabilitation. Future studies incorporating speech-language and quality-of-life measures are warranted to identify modifiable determinants and optimize outcomes across all populations.
